# Plant Volatiles, Rather than Light, Determine the Nocturnal Behavior of a Caterpillar

**DOI:** 10.1371/journal.pbio.0040164

**Published:** 2006-05-16

**Authors:** Kaori Shiojiri, Rika Ozawa, Junji Takabayashi

**Affiliations:** **1**Center for Ecological Research, Kyoto University, Kamitanakami, Otsu, Japan; **2**Japan Science and Technology Agency, Kawaguchi, Japan; Cornell UniversityUnited States of America

## Abstract

Although many organisms show daily rhythms in their activity patterns, the mechanistic causes of these patterns are poorly understood. Here we show that host plant volatiles affect the nocturnal behavior of the caterpillar Mythimna separata. Irrespective of light status, the caterpillars behaved as if they were in the dark when exposed to volatiles emitted from host plants (either uninfested or infested by conspecific larvae) in the dark. Likewise, irrespective of light status, the caterpillars behaved as if they were in the light when exposed to volatiles emitted from plants in the light. Caterpillars apparently utilize plant volatile information to sense their environment and modulate their daily activity patterns, thereby potentially avoiding the threat of parasitism.

## Abstract 2

The nocturnal feeding behavior of the caterpillar *Mythimna separata* is mediated by the volatiles released by the host plant, rather than by photoperiod, and may help the caterpillars avoid attack from parasitoids.

## Introduction

Photoperiod and the temperature are the most stable abiotic rhythms on the earth, and they are considered to be the principal exogenous factors that affect daily periodicity of behaviors of all organisms, particularly nocturnal feeders [ 1]. Nonetheless, many cues, including biotic factors, are available to foraging organisms, and each cue may have different potentials in determining daily activity patterns. For example, biotic factors such as predation or foraging ability based on sensory modalities would influence diel activity patterns. What is not well understood is how abiotic and biotic factors act in concert to regulate the daily activity patterns of foraging organisms. For example, studies of the effects of photoperiod on herbivorous insects typically place the insects on host plants, and behaviors are observed during different photoperiods. However, in addition to the light–dark cycle, the host plant is also potentially influencing the behavior of the insect because host plants are often not only food sources for herbivorous arthropods, but also their microhabitats. Host plants release odors, or volatiles, that have been shown to be influenced by photoperiod [ 2]. Thus, herbivorous arthropods are also confronted with a number of plant factors that potentially vary according to photoperiod and temperature, and it is critical to understand the role of each of these factors when determining the mechanisms regulating diel activity patterns.

Host plants are known to emit specific blends of volatiles in response to herbivory, and such volatiles are called herbivore-induced plant volatiles (HIPV) [ 3, 4]. Interestingly, HIPV show diurnal patterns [ 2, 5, 6]. For example, corn plants infested by larvae of the noctuid Spodoptera exigua emit S. exigua–induced plant volatiles ( S. exigua–IPV) that attract parasitic wasps [ 7]. S. exigua–IPV are composed of several monoterpenoids, sesquiterpenoids, green leaf volatiles, and the compound indole [ 7]. The production of S. exigua–IPV shows daily periodicity; emission increases in the daytime and decreases in the nighttime [ 2, 5]. *Cotesia marginiventris,* parasitic wasps of S. exigua larvae, are attracted to S. exigua–IPV in wind tunnel experiments [ 8], suggesting that the wasps actively search for S. exigua larvae during the day using S. exigua–IPV in the field. Maeda et al. (2000) also reported similar patterns in a tritrophic system consisting of kidney bean plants, the herbivorous mite *Tetranychus urticae,* and predatory mites *Phytoseiulus persimilis:* the production of T. urticae–IPV that attract the predatory mites increases during the day and decreases in the night [ 6]. These data suggest that host plants during the nighttime are an enemy-free space due to the lack of HIPV production, and such diurnal changes in HIPV production may play an important role in determining the day–night patterns of herbivorous insects.

Mythimna separata (Lepidoptera: Noctuidae) is a caterpillar that feeds on many graminaceous plant species. Corn plants infested by M. separata larvae emit M. separata–IPV that attract the parasitic wasp Cotesia kariyai (Hymenoptera: Braconidae) [ 9]. Sato et al. (1983) reported that C. kariyai was diurnal whereas M. separata larvae fed and were active primarily at night and are thus nocturnal [ 10]. When studying the day–night patterns of *M. separata* larvae, they used potted corn plants as food and observed the feeding and hiding behavior of the larvae on these plants [ 10]. However, the behavior of the larvae might have been affected by the day–night patterns in the production of infested corn plant volatiles, and they did not investigate this potentially confounding factor. Here we show for the first time that differences in volatiles from corn plants (either uninfested or infested by M. separata larvae) under light and dark conditions are critical factors affecting the daily periodicity of this herbivorous insect. In fact, we show that volatile cues were more important than light cues in modulating the hiding behavior of M. separata. We discuss possible explanations for the evolutionary and ecological significance of host plant volatiles as diel cues in a tritrophic context.

## Results

### Effects of Light on the Hiding Behavior of the Larvae

When offered only artificial diet, the numbers of larvae exhibiting hiding behavior were not different under the two light conditions ( Figure 1, black lines: generalized linear model, *p* = 0.754). This suggests that light alone was not sufficient to affect the hiding behavior of the larvae.

**Figure 1:**
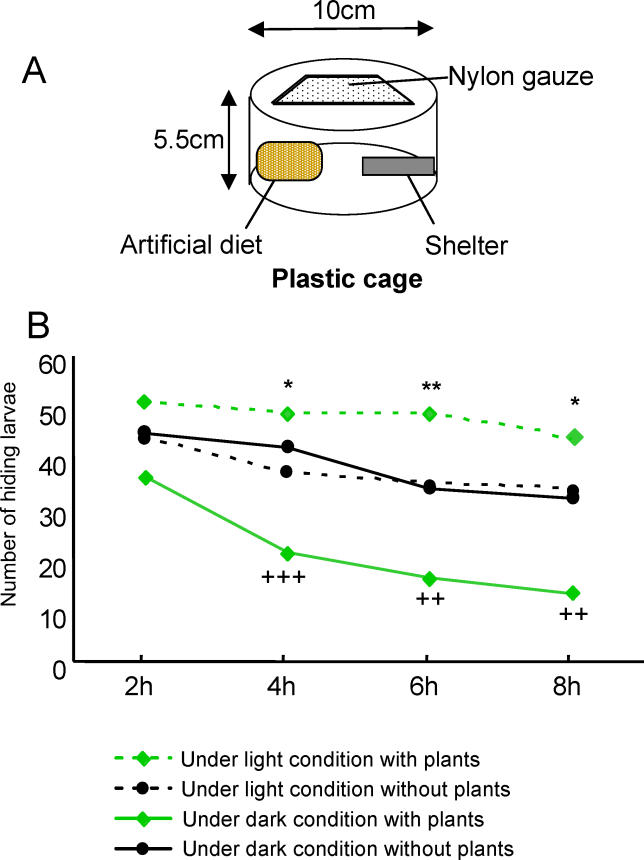
Effects of Light and Uninfested Corn Plants on the Hiding Behavior of M. separata (A) An illustration of the plastic cup in which the individual larvae are placed during the experiment. (B) The graph shows the number of larvae hiding ( *y*-axis) in 8 h ( *x*-axis). Asterisks (*) indicate the comparison between dashed lines (i.e., larvae under light with or without plants): A single asterisk (*) indicates 0.01 < *p* < 0.05, and double asterisks (**) indicate 0.001 < *p* < 0.01, by Fisher's exact probability test. Plus signs (+) indicate the comparison between solid lines (i.e., larvae under dark with or without plants): double plus signs (++) indicate 0.001 < *p* <0.01, and triple plus signs (+++) indicate *p* < 0.001, by Fisher's exact probability test.

### Effects of the Presence of Plants on the Hiding Behavior of the Larvae

The presence of plants affected caterpillar hiding behavior either positively or negatively relative to caterpillars without plants (generalized linear model, *p* < 0.0001). The interaction between light conditions and plant factors was also significant (generalized linear model, *p* < 0.0001).

Under the daytime light condition, the number of hiding larvae in the experiment with corn plants nearby was 12% (at 2 h), 21% (4 h), 25% (6 h), and 19% (8 h) higher than without corn plants (Fisher's exact probability test, at 2 h: *p* = 0.15, 4 h: *p* = 0.01, 6 h: *p* = 0.003, and 8 h: *p* = 0.03; Figure 1, black dashed line and green dashed line). Under dark conditions, on the other hand, the number of hiding larvae in the experiment with corn plants nearby was 14% (at 2 h), 35% (4 h), 30% (6 h), and 32% (8 h) lower than without corn plants (Fisher's exact probability test, at 2 h: *p* = 0.126, 4 h: *p* = 0.0002, 6h: *p* = 0.003, and 8 h: *p* = 0.0012; Figure 1, black line and green line).

### Effects of the Plant Volatiles on the Hiding Behavior of the Larvae

The experimental setups are illustrated in Figure 2A. The light conditions of the plants affected hiding behavior of the larvae (generalized linear model, Figure 2B: *p* < 0.0001 and Figure 2C: *p* < 0.0001). The light conditions of the larvae (generalized linear model, Figure 2B: *p* = 0.809 and Figure 2C: *p* = 0.416) and the interactions between the light conditions of the plants and larvae (generalized linear model, Figure 2B: *p* = 0.9391 and Figure 2C: *p* = 0.4722) were not significant.

**Figure 2:**
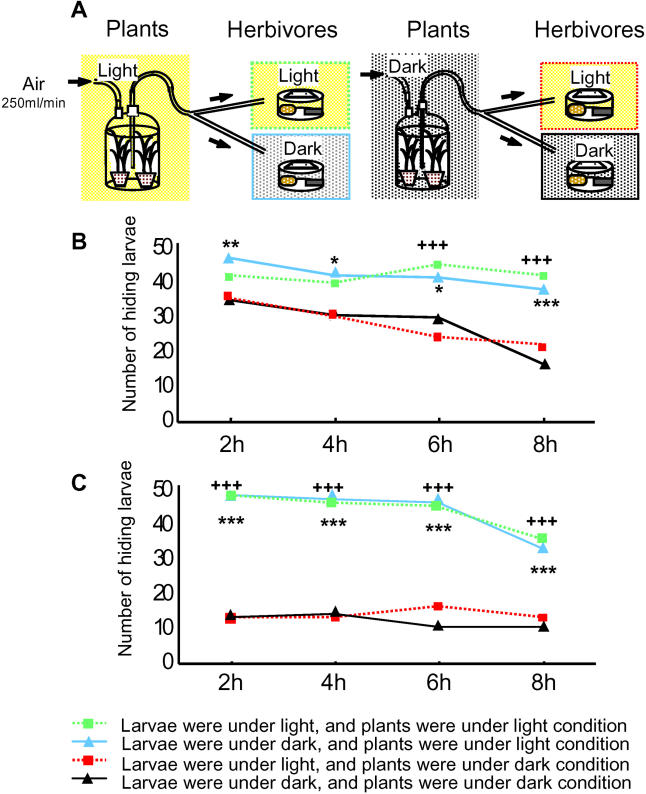
Effects of Light and the Volatiles from Plants under Either Light or Dark Conditions on the Hiding Behavior of M. separata (A) Experimental setups. Volatiles from corn plants were split into two airstreams and sent to incubators in which the larvae were kept under either dark or light conditions. (B) Number of larvae hiding when exposed to uninfested corn plant volatiles. (C) Number of larvae hiding when exposed to infested corn plant volatiles. For (B) and (C), asterisks (*) indicate the comparison between the blue line and the black line (i.e., the comparison of the effects of volatiles from uninfested/infested plants under light with those under dark on the hiding behavior of the larvae under dark): A single asterisk (*) indicates 0.01< *p* <0.05, double asterisks (**) indicate 0.001 < *p* < 0.01, and triple asterisks (***) indicate *p* < 0.001, by Fisher's exact probability test. Plus signs (+) indicate the comparison between the green line and the red line (i.e., the comparison of the effects of volatiles from uninfested/infested plants under light with those under dark on the hiding behavior of the larvae under light): Triple plus signs (+++) indicate *p* < 0.001, by Fisher's exact probability test.

When larvae were in the dark, the number of larvae hiding when experiencing volatiles from uninfested corn plants in light was 24% (at 2 h), 22% (4 h), 22% (6 h), and 42% (8 h) higher than those experiencing volatiles from uninfested corn plants in the dark (Fisher's exact probability test, at 2 h: *p*= 0.005, 4 h: *p* = 0.027, 6 h: *p* = 0.030, and 8 h: *p* < 0.0001; Figure 2B). Likewise, when larvae were in the light, the number of larvae hiding when experiencing volatiles from uninfested corn plants in light was 12% (at 2h), 18% (4h), 40% (6h), and 38% (8 h) higher than those experiencing volatiles from uninfested corn plants in the dark (Fisher's exact probability test, at 2 h: *p* = 0.24, 4 h: *p* = 0.083, 6 h: *p* < 0.0001, and 8 h: *p* < 0.0001; Figure 2B). These data indicate that it is not the light condition experienced by the larvae, but the volatiles from the corn plants (either under light or dark conditions) that affect the hiding behavior of the caterpillars.

The effects of volatiles on the larvae were similar on infested and uninfested plants. Irrespective of the light condition of the larvae, the number of larvae that hid when experiencing volatiles from infested corn plants in light was higher than larvae experiencing volatiles from infested corn plants in the dark (larvae under dark conditions: at 2 h (68%), 4 h (64%), 6 h (70%), and 8 h (44%); *p* < 0.0001 for each observation time; larvae under light conditions: at 2 h (68%), 4 h (64%), 6 h (56%), and 8 h (44%); *p* < 0.0001 for each observation time; Figure 2C).

## Discussion

Studies of day–night (or light–dark) patterns of herbivorous insects typically place the insects on host plants, and their behaviors are observed during different photoperiods. However, in this commonly used experimental design, light condition is confounded with other plant factors such as the emission of host plant volatiles. Sato et al. (1983) observed day–night behavioral patterns of M. separata larva on potted corn plants: The larva hid in a sheath of the host plant or underground during the day, and fed on the plants during the night [ 10]. Based on these data, they concluded that M. separata was nocturnal. However, while we were rearing M. separata larvae on an artificial diet, we found that they did not exhibit this day–night behavioral pattern ( Figure 1), suggesting that factors other than light could be important in determining the nocturnal behaviors that Sato et al. (1983) observed [ 10]. One important difference was that we used an artificial diet whereas Sato et al. (1983) used corn plants as food [ 10]. As clearly seen in Figure 1, factors from uninfested corn plants in the light enhance the daytime behavior of hiding in a shelter for M. separata larvae, whereas factors from plants in the dark enhance the nighttime behavior of leaving the shelter and feeding.

As corn plants show diurnal variation in the production of volatiles [ 5], we hypothesized that a difference in the blend of volatiles from corn plants that depends on light conditions affects the differences between daytime and nighttime behaviors of M. separata larvae. To test this hypothesis, we first provided volatiles from uninfested corn plants that were under either light or dark conditions, to the group of larvae in cups that were under either light or dark conditions ( Figure 2B). The data indicate that it is not the light condition of the larvae, but rather the volatiles from the uninfested corn plants that affect hiding behavior. We then repeated the experiments using corn plants infested by M. separata larvae as a source of volatiles. The larvae showed the same behavior as seen in the experiments using uninfested plants as an odor source: Irrespective of light conditions of the larvae, significantly more larvae hid when volatiles from infested plants under light conditions were offered than when volatiles under dark conditions were offered ( Figure 2C). It is important to note that the difference was more pronounced with volatiles from the infested plants ( Figure 2C). Plant volatiles are known to have numerous functions, such as attracting herbivores [ 11, 12] and/or carnivorous natural enemies of herbivores [ 3, 4, 7], repelling herbivores [ 13, 14], and attracting pollinators [ 15, 16], to name a few. Here, we suggest an additional function: Plant volatiles may affect the diurnal and nocturnal behavior of herbivores.

The circadian rhythms of insects have long been studied, and there are several potential causes for circadian rhythms [ 1]. We believe that the use of changes in host plant volatiles to alter diel patterns of feeding fits within a tritrophic framework. Insect parasitoids typically forage during the day [ 10, 17] and use diurnal plant volatile emissions as foraging cues [ 2, 5]. Consequently, caterpillars using day–night changes in the profiles of host plant volatiles to regulate their own activity rhythms could reduce the probability of parasitism by exploiting temporally available enemy-free space. Additional experiments are planned to determine (1) the compounds of plant volatiles that significantly affect the nocturnal behaviors of M. separata larvae either positively or negatively, and (2) if the modification of diel activity cycles by host plant volatiles is a widespread phenomenon in herbivorous insects, especially in those species that leave their host plants when not feeding.

## Materials and Methods

### Insects and plants.

M. separata was obtained from a culture reared at Tsukuba University in Tsukuba, Ibaraki, Japan. The insects were reared in our laboratory on artificial diet (Insecta LF, Nihon Nousan Kogyo, Yokohama, Japan) under conditions of 25 ± 2 °C, 24-h dark, 50%–70% relative humidity. This photoperiodic regime was selected to ensure that there would be no photoperiodically entrained diel periodicity at the time the different assays were carried out.

Potted corn plants ( Zea mays L. cv. Royal Dent) (three plants per pot) were grown in a growth chamber (25 ± 2 °C, 16:8-h light:dark), and 10-d-old plants were used in all experiments.

### Effects of light on the hiding behavior of the larvae.

Based on the data by Sato et al. (1983), M. separata larvae feed and are active primarily at night [ 10]. They hide in the sheath of a host plant or under the ground during the day. Here, we focused on hiding as the criterion for diurnal behavior. We placed 57 third-stadium larvae in individual plastic cups (20-cm diameter and 6-cm height) with filter paper shelters (folded in accordion manner: 4 × 4 cm), each with a piece of artificial diet (ca. 7 g). The top of each cup had a 5 × 5 cm nylon gauze window ( Figure 1A). The cups were kept in an incubator under either 6500 lux light conditions or dark conditions for 8 h at 25 ± 2 °C with 50%–70% relative humidity. We observed larval behavior every 2 h, to determine whether or not larvae were hiding in the shelters.

### Effects of the presence of plants on the hiding behavior of the larvae.

To study the effects of the host plants on the feeding behavior of the larvae, we conducted similar experiments as above, only with uninfested corn plants. Uninfested corn plants are those that have never experienced herbivory. We placed six pots of uninfested corn plants, each containing three plants of circa 30 cm height per pot, around the 57 cups containing third-stadium larvae in an incubator of the same climate conditions as above.

### Effects of the plant volatiles on the hiding behavior of the larvae.

To test the hypothesis that host plant volatiles affect the diurnal feeding behavior of the caterpillars, we conducted similar experiments as above, with the addition of plant volatiles. Four pots of three plants each were placed into a 7,200-ml plastic separable flask. Volatile flow emitted from the plants (under either light [6,500 lux] or dark conditions) were collected in a 250-ml/min flow stream that was split into two, and each flow was directed to an incubator containing 50 third-stadium larvae. These larvae were housed individually in cups for 8 h under either light (6500 lux) or dark conditions ( Figure 2; 25 ± 2 °C, 50%–70% relative humidity). We checked whether the larvae were hiding in the shelters every 2 h. We used both intact and infested corn plants as the odor source. To obtain infested plants, we placed ten third-stadium M. separata larvae in each pot on the corn plants. After 18 h, the larvae and their feces were removed, leaving behind a damaged area of circa 10% of the total leaf surface. In these experiments, all caterpillars were subjected to the same airflows; however, we independently tested day and night volatiles from infested and uninfested plants, and obtained similar results.

### Statistics.

We analyzed the data in Figure 1 using generalized linear models and binomial errors with the software program R 1.7.0 for Windows [ 18] in order to test the hypothesis that it was not the light condition experienced by the larvae, but factors from intact corn plants that affected the hiding behavior of the caterpillars. Differences in hiding behaviors between different conditions of larvae (e.g., light conditions of larvae, and presence or absence of intact plants nearby) were analyzed. We also analyzed the data in Figure 2A and 2B with R to test the hypothesis that it was the volatiles from corn plants that affected the hiding behavior of the larvae and not the light condition experienced by the larvae. Differences in hiding behavior between different conditions of larvae (e.g., light conditions of larvae and the light condition of the plants) were analyzed. Then, the numbers of larvae hiding in different treatments were compared with Fisher's exact probability test.

## Abbreviations

HIPV: herbivore-induced plant volatiles
–IPV: –induced plant volatiles
